# Medical cannabis legalization and the use of illicit drugs, alcohol, and tobacco

**DOI:** 10.1186/s42238-025-00324-5

**Published:** 2025-09-09

**Authors:** Hana Al Hallaj, Zahraa Barakat

**Affiliations:** 1 Technische Universität Berlin , Building H, Space H4136A, Straße des 17. Juni 135, Berlin, 10623 Germany; 2 Department of EconomicsMA in Applied Economics, Lebanese American University, P.O. Box: 13-5053, Beirut, Lebanon

**Keywords:** Medical cannabis, Innovation, Sales, Marijuana, Alcohol, Tobacco, Amphetamines, Fixed effect model, Dynamic DiD, Difference in differences, Staggered adoption, Average treatment effect

## Abstract

**Purpose:**

Amidst the global shift toward cannabis legalization, this study examines medical cannabis (MC) sales as an indicator of economic activity and innovation. It explores associations between MC sales, and variables including tobacco use, alcohol consumption, amphetamine, cocaine and cannabis prevalence, and gross domestic product (GDP), using a fixed effects (FE) panel regression model. It also evaluates associations between cannabis legalization and MC sales over time using a dynamic Difference-in-Differences (DiD) approach with multiple time periods.

**Methods:**

Panel data from 20 countries, including 14 with legalized medical cannabis and 6 without, are analyzed. The dynamic DiD approach estimates the average association for legalizing countries across multiple time periods, enabling comparisons of post-legalization outcomes with those of non-legalizing countries.

**Results and conclusions:**

The findings indicate a strong negative association between tobacco use and MC sales, while cannabis consumption shows a positive association with MC markets. Amphetamine use is negatively associated with MC sales, suggesting substitution dynamics. Legalization is associated with an average annual increase of 26.06 tons of MC sales in legalizing countries. Event study estimates confirm a sustained growth trajectory in MC sales following legalization. A robustness check, which excludes the United States—a major outlier in market size—yields a slightly lower average effect of 20.05 but still supports the persistent market expansion.

Given the ecological nature of the design, these results should be interpreted as population-level associations rather than individual-level causal effects. Nonetheless, they highlight the potential economic relevance of cannabis legalization in expanding regulated markets and reshaping consumer behavior. The study contributes to debates on legalization, public health, and economic policy by providing empirical evidence on the associations between legal reforms and market dynamics.

## Introduction and theoretical background

### Introduction

Research on Medical Cannabis Legalization (MCL) has largely focused on its health and social effects, particularly in the United States, where studies have examined its impact on adolescent cannabis use (Harper et al., 2012); Hasin et al., 2015; Wall et al.,  2011); Sarvet et al., 2018) and cannabis use disorder (CUD) (Hoch et al. 2024). While some research has explored MCL’s relationship with public health outcomes, its economic and market implications remain understudied. The novelty of the cannabis industry and legal inconsistencies across countries have constrained data availability, leaving critical gaps in understanding how MCL affects broader substance markets, including alcohol, tobacco, and illicit drugs. Little is known about how MCL shapes international cannabis markets, especially in terms of sales, innovation, and trade. As legalization expands globally, understanding its economic effects and interactions with other substance markets is increasingly important for policy design. 

This study aims to fill these gaps by applying a dynamic DiD regression approach across 20 countries, including 14 with legalized medical cannabis and 6 without. It investigates the determinants of medical cannabis sales and assesses whether MCL acts as a substitute for or complement to other substances, analyzing trends in in gross domestic product (GDP), alcohol, tobacco, cannabis, amphetamines, and cocaine prevalence. Additionally, it examines the causal impact of MCL over time, using MC sales as a proxy for innovation.

### Theoretical background

#### Sales as an innovation proxy

Only a small part of the literature uses innovative sales as a proxy for innovation (Prencipe et al. (2022), Jensen and Webster (2009), Kleinknecht et al. (2002), Gottschalk and Janz (2001), Bass (1969), von Hippel (2005). Prencipe et al.’s findings in 2022 suggest that evaluation studies of innovation support programs should be planned to capture not only input but also output effective measures of innovation, such as those related to the economic returns of innovations. In fact, the percentage of sales derived from new products is often used as a proxy for the economic returns of the innovation process, highlighting the critical role of economies of scale in driving innovation outcomes.

Jensen and Webster 2009 looking at the relationship between innovation and proxies from an economic and productivity perspective for firms, references Kleinknecht et al. (2002) and suggests that in absolute terms, and when taking a broad range of firms, there were clear differences between two groups of innovation indicators – R&D (Research and Development)/innovation expenditure/patent applications on the one hand and sales of new products on the other hand. Gottschalk and Janz (2001) found a positive long–run effect of R&D on markets’ sales concentration suggesting that innovative activity is measured not only by R&D expenditure but by the share of R&D expenditure in total sales.

The Bass diffusion model, developed by Frank Bass, describes the adoption process of new products within a population (Bass, F. M. 1969). In this model, sales figures represent the rate of new product adoption, thereby serving as an indirect measure of innovation diffusion. Furthermore, research on user innovation, as investigated by Eric von Hippel (von Hippel, E. 2005), considers sales metrics to evaluate the impact of consumer-developed innovations on markets, highlighting the significance of sales as an indicator of innovation diffusion and acceptance.

Al Hallaj et al. (2025) studied patents as a proxy for innovation to investigate some of the main criteria for cannabis innovation on a global scale. The investigation centered on crucial determinants such as publications, R&D activities, and clinical trials within the cannabis innovation landscape. However, it is fair to state that patent applications are only bound by innovations that are patentable inventions and hence will not account for a comprehensive output measure of innovation. This paper will tackle the commercialized outputs of innovation, in this case MC sales is the experimented dependent variable.

While sales figures are widely used as a proxy for innovation output and diffusion, they do have limitations. As discussed by Jensen and Webster (2009), such indicators may also reflect broader contextual factors, including national regulations, supply-side constraints, and prevailing cultural attitudes toward product adoption. Thus, while sales volume is employed in this study as a practical indicator of commercialized innovation, it should be interpreted with caution, recognizing that it may also capture external institutional and structural influences beyond the innovation process itself.

#### Legalization and substance use

In the United States, several studies have examined the dynamics of substance use following the enactment of MCL in states, comparing them with states where such legislation has not been implemented Cerdá et al. (2018), Weinberger et al. (2022), Kaufmann et al. (2021), Coley et al. (2021), Anderson et al. (2014). These investigations have yielded heterogeneous results, as exemplified by the research conducted by Cerdá et al. (2018). Notably, Cerdá and colleagues found that, following MCL enactment, the prevalence of cannabis use, binge drinking, cigarette use, non-medical use of opioids, amphetamines, tranquilizers, and any non-marijuana illicit drug use decreased among young adolescents. A plausible explanation for this decline lies in changes in the relative prices of these substances, particularly in the context of marijuana's changing legal status. However, it is important to note that the picture differs among older adolescents, where states legalizing MC observed an increase in non-medical prescription opioid and cigarette use following MCL enactment. These findings underscore the complexity of the impact of MCL on substance use behaviors, with age and substance-specific factors contributing to the heterogeneity of outcomes.

In 2022, Weinberger et al. conducted a comprehensive investigation into the effects of recreational and medical cannabis laws (RCL and MCL) on patterns of cannabis and cigarette consumption in the United States, employing a rigorous DiD analytical approach. Their findings also revealed nuanced outcomes, particularly with respect to MCL. While MCL was associated with an overall increase in the co-use of cigarettes and cannabis, a notable exception was observed among individuals under the age of 18, where co-use declined. A salient observation from the study was the concurrent consumption of these substances, often through joint usage, intensifying the inhalation of tetrahydrocannabinol (THC). Furthermore, the investigation posited that the phenomenon of substitution, wherein cannabis is chosen as an alternative to cigarettes due to perceptions of reduced addictiveness, harm, and social acceptability, contributes to the observed trends. In comparison, RCL was associated with an overall decrease in cigarette-only use.

Goodwin et al. (2018) conducted a nationally representative cross-sectional study using data from the National Survey on Drug Use and Health (NSDUH), which surveys individuals aged 12 and older in the United States. Their findings indicate that daily cannabis use is most prevalent among cigarette smokers, with increases observed among current, former, and never smokers over the past decade. Notably, the most rapid increases in daily cannabis use were found among youth and female cigarette smokers.

On a discussion about potential impacts on opioid on states that have legalized MC and those that did not, Kaufmann et al. (2021) found that, contrary to this previous work, opioid overdoses did not decrease in the years subsequent to states adopting MC as compared to states that did not. In fact, states that adopted MC had significantly greater overdose slopes than those that did not. MC states had higher rates of opioid overdoses. Although there was no decrease in association with MC introduction, these results were confounded by states without MC having lower overdose reporting quality. Medicaid expansion was also more common in states with MC.

From an RCL perspective, Coley et al. (2021) conducted an assessment utilizing a national sample of high school students. Their findings indicated minimal associations between the legalization of recreational marijuana and adolescent substance use. Specifically, they observed modest declines in the frequency of marijuana use among those who use it, along with slight increases in the likelihood of e-cigarette use.

Anderson et al. (2014), in their argument, proposed that the evolving cannabis markets, transitioning from illegal markets with lax age restrictions to legal markets with stricter enforcement of the minimum age of 21, might contribute to reduced adolescent access to cannabis products. Alternatively, changes in adolescent substance use patterns may be attributed to interpersonal factors. Earlier research, focusing on minor declines in adolescent marijuana use after MCL, posited that shifting attitudes and heightened awareness regarding marijuana's accessibility could alter parental supervision, thereby limiting youth substance use.

It is important to note that the connection between the initiation of e-cigarette use and subsequent cannabis use among adolescents has been documented, raising concerns about potential long-term repercussions of recreational cannabis legalization on adolescent marijuana use. Worth mentioning is that the item assessing e-cigarette use does not specify whether it pertains to smoking tobacco or marijuana, both of which could be consumed through this modality.

Comparing cannabis and alcohol, Anderson et al. (2014) found a positive correlation between marijuana and alcohol use in cross-sectional analyses, illustrating evidence indicating a notable decline in marijuana use when individuals reach the minimum legal drinking age. This suggests that, for young adults, there may be a substitution effect wherein marijuana is utilized as an alternative to alcohol.

Cannabis use patterns differ notably between medical and non-medical users. Medical patients typically consume standardized doses under healthcare supervision to manage conditions like chronic pain or epilepsy (Hoch et al. 2024). In contrast, recreational users often seek psychoactive effects through high-THC products, which are associated with increased risks of cognitive impairment, addiction, and mental health disorders (Hoch et al. 2024).

In contrast to Cerdá et al. (2018), Coley (2021), and Anderson (2014), which primarily examine cannabis use among adolescents, this study focuses on adult population patterns related to cannabis consumption, illicit drug use, tobacco, and alcohol. This distinction is driven by the availability and completeness of United Nations (UN) data sources, which provide more extensive and reliable information on adults compared to adolescents. Furthermore, unlike Weinberger et al. (2022) and Coley (2021), which explore the impact of Recreational Cannabis Legalization (RCL), this study specifically analyzes Medical Cannabis Legalization (MCL) sales data. This approach is justified by the limited availability of RCL data at the global level, as recreational cannabis markets remain underdeveloped or unregulated in many countries. While some countries in the sample have also legalized recreational cannabis, this study does not consider the implications of recreational legalization. The analysis is limited to medical cannabis laws, and all discussions of legalization and cannabis sales are to be understood within that context.

This study extends U.S.-focused research to a global comparison, examining medical cannabis use across countries and its relationship with tobacco, alcohol, and illicit drugs. By doing so, it contributes new insights to the literature on international substance use patterns and the broader implications of medical cannabis legalization.

## Methodology

### Data description

This study synthesizes data from multiple sources to analyze the factors influencing MC sales and to evaluate the impact of regulatory environments on market dynamics. The dependent variable is the annual sales volume of medical cannabis (measured in tons), sourced from the Market Data Forecast database.

- The explanatory variables outlined below capture patterns of substance use and key economic conditions, both of which may influence MC sales. Alcohol Consumption per Capita, Prevalence of Cannabis, Cocaine, and Amphetamine Use (percentage of the population aged 15–64), sourced from the United Nations Office on Drugs and Crime (UNODC). These variables reflect broader substance use behaviors, which may interact with MC demand through complementarity or substitution effects (Subbaraman 2016; Gunn & Smith 2022).
- Tobacco Smoking Prevalence (percentage of current smokers in the adult population), compiled from the World Health Organization (WHO). Research indicates a significant overlap between nicotine and cannabis use, with studies showing that approximately 40% of medical cannabis patients also consume it with nicotine, suggesting that tobacco use may be associated with MC demand (Bridgeman et al. 2022). Additionally, higher tobacco use rates may reflect social acceptance of cannabis consumption, influencing MC adoption at the national level.
- GDP (measured in USD and expressed in natural logarithm to adjust for scale differences), sourced from the World Development Indicators (WDI) database. GDP serves as a proxy for economic capacity, influencing both affordability and accessibility of MC.

The dataset includes 20 countries, 14 of which have legalized medical cannabis at different points in time, while the remaining 6 have not implemented legalization. This variation allows for both a comparative analysis of MC sales trends and an evaluation of legalization effects over time. Table 1 provides an overview of legalization status and respective years of legalization, with data sourced from official government portals.

**Table 1:** Countries under review and their MC legalization status

Country	Legalized MC	Date	Source^a^
Australia	Yes	2016	https://www.tga.gov.au/
Brazil	Yes	2019	https://antigo.anvisa.gov.br/
Canada	Yes	2001	https://laws-lois.justice.gc.ca/
China	No	-	NA (Not Available)
Denmark	Yes	2018	https://laegemiddelstyrelsen.dk/
France	No	-	NA
Germany	Yes	2017	https://www.bundesgesundheitsministerium.de/
India	No	-	NA
Israel^b^	Yes	1990	https://www.gov.il/
Italy	Yes	2013	https://def.finanze.it/
Japan	No	-	NA
Mexico	No	-	NA
Netherlands	Yes	2003	https://english.cannabisbureau.nl/
Poland	Yes	2017	https://sejm.gov.pl/
Portugal	Yes	2018	https://diariodarepublica.pt/
Spain	No	-	NA
Switzerland	Yes	2019	https://www.swissmedic.ch/
Thailand	Yes	2018	https://en.fda.moph.go.th/
United Kingdom	Yes	2018	https://www.gov.uk/
United States^c^	Yes (38 states)	2010 (average)	https://cannabis.ca.gov/, https://kymedcan.ky.gov/

Source: Data compilation

^a^Legalization year and policy status compiled from official national government publications. The “Source” column lists the official website of the issuing entity

^b^No governmental source found specifying date of MC legalization for Israel. However, date has been reported on The Jerusalem Post https://www.jpost.com/

^c^California (1996) and Kentucky (2025) are cited only to illustrate the legalization year range across the 38 states

### Model specification and design

To investigate the factors that influence MC sales, we use a linear regression model, specifically a fixed-effects (FE) panel regression, with annual sales volume of medical cannabis as continuous dependent variable. This approach accounts for time-invariant unobserved variability across countries, such as cultural attitudes, healthcare facilities, and regulatory frameworks, all of which may influence MC sales. By focusing on within-country fluctuations across time, the model isolates the impact of time-varying factors, resulting in a more accurate estimate of the link between MC sales and its primary determinants.

To address missing data and gaps in certain years, interpolation and extrapolation techniques were employed to construct a more comprehensive and robust dataset. Additionally, Variance Inflation Factors (VIFs) were calculated to assess the presence of multicollinearity among the explanatory variables. With an average VIF score below 10 and all VIF values under 5, the results indicate that multicollinearity does not pose a concern, thereby confirming the model’s reliability.

The FE Model is specified as follows:

1 $$Sales_{it}=a_i+\beta X_{it}+\varepsilon_{it}$$

where:

- Sales(it) is the medical cannabis sales variable for country i at year t, measured in tons.
- αi represents the country-specific intercept, capturing time-invariant characteristics unique to each country.
- β denotes the vector of coefficients for the independent variables.
- Xit​ is the vector of independent variables for country i at year t- which includes:
  - ◦ Alcohol Consumption per Capita- Liters of pure alcohol per person
  - ◦ Prevalence of Cannabis, Cocaine, and Amphetamine Use (%)
  - ◦ Tobacco Smoking Prevalence (%)
  - ◦ GDP (expressed in natural logarithm)
- ϵit​ is the error term for country i at year t

To evaluate the impact of cannabis legalization on MC sales, this study employs a DiD approach with staggered treatment timing. Rather than relying on the traditional two-period DiD framework, our analysis employs a dynamic DiD approach that accommodates multiple time periods and staggered treatment adoption across units. This methodology captures treatment effects through a series of interaction terms between treatment status and time relative to treatment, thereby allowing for the estimation of dynamic effects that evolve across different post-treatment periods. Given that legalization occurred in different years across multiple countries, a standard DiD model assuming a single treatment year would fail to fully capture these dynamics. Instead, the analysis adopts a generalized DiD framework that accounts for each country’s unique legalization timeline, comparing pre- and post-legalization periods relative to each country’s specific treatment year. This approach not only allows for a more flexible estimation of policy effects but also captures potential heterogeneity in outcomes by considering variations in institutional contexts, enforcement mechanisms, and baseline economic conditions.

To estimate treatment effects across multiple periods, the study applies the Callaway and Sant’Anna (2022) estimator, a robust method tailored for staggered policy adoption. This approach accounts for group-specific treatment effects over time, reflecting differences in policy impact across various settings. It also facilitates an event study analysis to examine the dynamic effects of legalization both before and after its implementation. Prior to estimating treatment associations, we validated the parallel trends assumption by examining pre-legalization trajectories of MC sales for treatment and control groups through event study plots. The absence of statistically significant pre-treatment differences supports the validity of this assumption, allowing for more reliable interpretation of post-legalization associations. By appropriately controlling for never-treated units, the analysis mitigates potential biases in comparisons, ensuring more robust and reliable estimates.

The empirical model adopts a Group-Time Average Treatment Effect on the Treated (ATTgt) framework, wherein treatment effects are estimated for each cohort g (i.e., countries grouped by the year they legalized medical cannabis) and each time period t relative to treatment. This more granular specification allows us to observe how the effect of legalization evolves over time across different groups. When aggregated, these estimates yield the overall ATT, summarizing the average impact of legalization across all treated countries.

The estimation is conducted using the following specification:

2 $$Sales_{it}=\alpha i+\lambda t+\delta\left(g,\;t\right)Dit\left(g\right)+\epsilon it$$

where:

- Sales(it) is the medical sales variable for country i at year t
- αi​ captures country fixed effects,
- λt ​ captures time fixed effects,
- Dit(g)​ is an indicator for whether country i was treated in group g and year t
- δg,t​ is the group-time ATTgt, capturing the treatment effect for group g in year t.
- ϵit​ is the error term

Notably, the estimation employs Doubly Robust Inverse Probability Weighting (DRIPW) to correct for selection bias, combining Inverse Probability Weights (IPW) and Regression Adjustment (RA). IPW adjusts for differences in treatment probabilities, ensuring comparability, while RA models potential outcomes to enhance efficiency and robustness. This dual approach improves the credibility of treatment effect estimates by reducing bias and accounting for pre-treatment differences.

The sample size was reduced to 19 countries, excluding Israel, which legalized MC in 1990, due to the absence of a valid comparison group. Since the dynamic DiD requires pre-treatment data and a control group, early legalization precludes meaningful comparisons. Including Israel could introduce bias as the lack of a clear baseline and the extended gap between treatment and control groups would undermine the validity of the analysis. In contrast, although California legalized medical cannabis in 1996, the United States was treated as a single national unit in our analysis, not at the state level. We applied an average national treatment year of 2010, reflecting broader legalization trends across U.S. states. This aggregation allows the U.S. to be retained in the model without violating comparability assumptions, unlike the case of Israel. As the data spans 2000–2023, always-treated countries (treated before 2000) are excluded since the Callaway-Sant’Anna (2022) estimator relies on comparisons with either never-treated or later-treated units.

During the initial stage of grouping countries by the timing of their medical cannabis legalization, we encountered challenges related to limited comparability. The estimator proposed by Callaway and Sant’Anna (2022) requires sufficient overlap between treated and untreated units to generate valid ATT estimates in staggered adoption settings. This posed a problem for early adopters, such as the Netherlands (2003), which lacked comparable untreated units in the years following legalization. Similarly, the United States (2010), with significantly higher levels of medical cannabis sales, and Italy (2013), surrounded by quickly treated peers, had limited comparability with the few untreated countries that remained, most of which had no observable cannabis activity.

To mitigate these limitations and preserve the integrity of the analysis, we adopted a pragmatic grouping approach. Countries that legalized early and in close succession, such as Canada (2001) and the Netherlands (2003) were grouped together. Meanwhile, the United States (2010) and Italy (2013) were merged with Australia (2016), which marked the beginning of a second wave of legalization. Germany and Poland (2017) formed a distinct group, as did the United Kingdom, Denmark, Thailand, and Portugal (2018). Lastly, Brazil and Switzerland (2019) were treated as a final cohort. This strategy, which reflects guidance from Callaway and Sant’Anna, helped ensure that group-level ATT estimates were both statistically valid and methodologically sound, while improving model performance and preserving the richness of temporal trends in treatment effects.

As a robustness check, we perform a sensitivity analysis by re-estimating the staggered DiD model (Callaway & Sant’Anna, 2022) after excluding the United States from the sample. Given the U.S.'s dominant role in global cannabis patenting and its unique legal and institutional context, its inclusion may disproportionately influence the estimated treatment effects. With a significant share of the global medical cannabis market, the United States may produce results that are not entirely indicative of other nations due to its distinct legal environment, size, and well-established cannabis industry. This analysis allows us to assess whether our findings are driven by the U.S. case or are consistent across other countries, thereby testing the stability and generalizability of the estimated ATT.

All statistical analyses were conducted at a 5% significance level (α = 0.05), with p-values below this threshold considered statistically significant. Data management, descriptive statistics, and econometric estimations were performed using Stata 18.

## Results and findings

The FE regression model examining the determinants of medicinal cannabis sales is statistically significant (F = 14.88, *p* = 0.000), confirming that at least one independent variable has a significant influence on sales. The model captures 40% of the variation within countries over time, highlighting the relevance of time-varying components (within R^2^ = 0.400). Furthermore, the model suggests a strong fit, as indicated by the Akaike Information Criterion (AIC = 1371.616) and the Bayesian Information Criterion (BIC = 1393.142), confirming the robustness of the estimated effects.

The findings indicate that cannabis use prevalence is positively and significantly associated with a MC sales, a one percentage point increase in cannabis use is linked to an estimated increase of 3.971 tons in annual MC. Conversely, amphetamine use (β = −10.485) and tobacco smoking prevalence (β = −8.581) exhibit a statistically significant negative MC sales. All results are deemed statistically significant at the 5% level (Table 2).

**Table 2:** Adjusted fixed effects linear regression model

MC sales (tons)	Coefficient	Std. Error	T value	95% CI	*P* value	Significance
GDP (log)	6.20	17.55	0.35	(−28.52, 40.91)	0.73	
Alcohol consumption (liters per capita)	5.58	3.40	1.64	(−1.14, 12.31)	0.10	
Tobacco smoking prevalence (%)	−8.58	1.93	−4.46	(−12.39, −4.77)	0.00	***
Cocaine use prevalence (%)	−0.06	5.08	−0.01	(−10.11, 9.99)	0.99	
Cannabis use prevalence (%)	3.97	0.92	4.34	(2.16, 5.78)	0.00	***
Amphetamine use prevalence (%)	−10.49	4.53	−2.32	(−19.43, −1.54)	0.02	**
Constant	−57.23	526.40	−0.11	(−1098.36, 983.89)	0.91	

^***^ *p* < 0.01, ** *p* < 0.05, * *p* < 0.1

The ATT is estimated by the Dynamic DiD model, which quantifies the causal impact of medical cannabis legalization solely on the countries that have implemented it. The findings suggest that the legalization of cannabis resulted in an average increase of 26.056 tons in medical cannabis sales in countries that enacted legalization. This effect is statistically significant at the 5% level, validating the robustness of the results.

The event study analysis confirms a clear and statistically significant increase in medical cannabis sales, measured in tons, following legalization (Table 3). On average, legalization resulted in an increase of 31.71 tons, highlighting a substantial and lasting impact. The initial effect was observed in the first year, with sales rising by 3.17 tons. This growth continued steadily, reaching 15.49 tons in the second year and 30.89 tons in the third year, signaling sustained expansion. The impact peaked later, with sales surging by 104.68 tons, reflecting a sharp rise in demand over time. Long-term estimates further support this trend, as sales remained significantly elevated even years after legalization. For instance, twelve years of post-legalization, sales increased by 15.01 tons, and by the twenty-second year, the effect reached 43.47 tons, indicating a structural shift in market demand.

**Table 3:** ATT by periods before and after treatment event study: dynamic effects (including US)

Time period (Years since legalization)	ATT	Std. Error	95% CI	*P* value
Pre average	0	(omitted)		
Post average	31.71	6.24	(19.49, 43.94)	< 0.001
Year 0	3.17	0.99	(1.23, 5.12)	< 0.001
Year 1	9.92	3.12	(3.80, 16.04)	< 0.001
Year 2	15.49	5.02	(5.66, 25.32)	< 0.001
Year 3	30.89	9.79	(11.71, 50.07)	< 0.001
Year 4	34.72	12.48	(10.26, 59.19)	0.01
Year 5	53.34	19.76	(14.62, 92.06)	0.01
Year 6	50.98	27.55	(−3.02, 104.98)	0.06
Year 7	104.68	44.60	(17.26, 192.10)	0.02
Year 9	10.87	2.53	(5.91, 15.83)	< 0.001
Year 12	15.02	2.48	(10.16, 19.87)	< 0.001
Year 14	17.78	2.44	(13.00, 22.56)	< 0.001
Year 15	21.64	4.09	(13.62, 29.66)	< 0.001
Year 16	25.50	5.75	(14.23, 36.77)	< 0.001
Year 17	29.36	7.40	(14.85, 43.87)	< 0.001
Year 18	31.96	8.27	(15.76, 48.17)	< 0.001
Year 19	34.57	9.13	(16.67, 52.46)	< 0.001
Year 20	37.49	10.38	(17.15, 57.84)	< 0.001
Year 22	43.48	12.97	(18.06, 68.89)	< 0.001

To further isolate the effects of legalizing medical cannabis across a more balanced group of economies, we re-estimated the Callaway & Sant'Anna (2022) DiD) model excluding the U.S. The results show a decreased but still statistically significant ATT, with the overall ATT dropping from 26.06 to 20.05.

Table 4 illustrates the average annual sales volume of medical cannabis following legalization declines slightly from 31.71 to 26.48 tons but remains considerable. The initial treatment effect remains positive and statistically significant (2.27 tons, *p* < 0.001), indicating an early increase in sales. Over time, the effect strengthens, peaking seven years after legalization at 50.66 tons (*p* < 0.001). This impact remains long-lasting, with sales still 43.47 tons higher after 22 years.

**Table 4:** ATT by periods before and after treatment event study: dynamic effects (excluding US)

Time period (Years since legalization)	ATT	Std. Error	95% CI	*P* value
Pre average	0	(omitted)		
Post average	26.48	4.65	(17.36, 35.60)	< 0.001
Year 0	2.28	0.53	(1.24, 3.31)	< 0.001
Year 1	8.12	2.87	(2.49, 13.75)	0.01
Year 2	13.29	4.93	(3.63, 22.96)	0.01
Year 3	24.25	8.46	(7.67, 40.84)	0.00
Year 4	23.29	7.09	(9.41, 37.18)	0.00
Year 5	36.15	13.66	(9.38, 62.93)	0.01
Year 6	50.98	27.55	(−3.02, 104.98)	0.06
Year 7	50.67	9.98	(31.10, 70.23)	< 0.001
Year 9	10.87	2.53	(5.91, 15.83)	< 0.001
Year 12	15.02	2.48	(10.16, 19.87)	< 0.001
Year 14	17.78	2.44	(13.00, 22.56)	< 0.001
Year 15	21.64	4.09	(13.62, 29.66)	< 0.001
Year 16	25.50	5.75	(14.23, 36.77)	< 0.001
Year 17	29.36	7.40	(14.85, 43.87)	< 0.001
Year 18	31.96	8.27	(15.76, 48.17)	< 0.001
Year 19	34.57	9.13	(16.67, 52.46)	< 0.001
Year 20	37.49	10.38	(17.15, 57.84)	< 0.001
Year 22	43.48	12.97	(18.06, 68.89)	< 0.001

This robust test confirms that the results are not driven by a single outlier economy, reinforcing the reliability of our findings. The persistence of treatment effects across varying economic contexts supports their broader applicability and significance.

## Discussion

### Discussion of results

The findings suggest a strong potential substitution effect between MC and tobacco consumption. Specifically, a one-percentage-point increase in tobacco use is associated with an estimated 8.581 ton decrease in annual MC sales, aligning with prior research suggesting these substances can act as economic substitutes (Cameron & Williams 2001; Wen et al. 2015; Bridgeman et al. 2022).

Likewise, cannabis use appears to be complementary to MC sales, a one-percentage-point increase in cannabis prevalence among individuals aged 15–64 is associated with an additional 3.971 tons of medical cannabis sales per year, reinforcing the complementarity hypothesis (Smart et al. 2017; Bradford & Bradford 2017). In contrast, a one-percentage-point increase in amphetamine use among the same age group is associated with a 10.485 tons annual decline in MC sales, which may reflect substitution dynamics due to medical need differentiation or regulatory restrictions limiting co-use.

These findings contribute to the literature on substitution and complementarity between MC and other substances, emphasizing substance-specific dynamics less explored in prior work (e.g., Cerdá et al. 2020).

The results from the Dynamic DiD regression indicate a substantial and statistically significant association between legalization and increased MC sales. Specifically, after controlling for time trends and country-specific FE, countries that legalized MC were associated, on average, with an annual increase of approximately 26.06 tons in sales compared to those that did not legalize.

The event study analysis shows dynamic treatment associations, with a sustained increase in MC sales in the periods following legalization. The robustness of our findings is strengthened by validation of the parallel trends assumption, as pre-treatment trajectories of MC sales were statistically indistinguishable between treatment and control groups. This supports confidence that the observed post-legalization associations are not driven by pre-existing differences in trends. These results highlight the sizable and lasting potential impact associated with legalization, reflected in both immediate and long-term growth in the MC market.

When excluding the U.S., the results continue to show significant increases in MC sales, though with reduced magnitude. This shows that the U.S may have previously amplified the estimated associations due to its larger market size, higher demand elasticity, and more advanced cannabis infrastructure.

Nonetheless, the event study results confirm that legalization is associated with a robust and persistent influence on MC sales across countries, even in the absence of U.S. data, reinforcing its role as a proxy for cannabis- related innovation output.

### Limitations

A key limitation of this study is its ecological study design, which relies on aggregate-level data rather than individual-level observations. While this approach allows for the identification of broad patterns and trends in medical cannabis sales and its relationship with illicit substance use, tobacco, and alcohol consumption across countries, it is subject to ecological fallacy—the risk that associations observed at the population level may not accurately reflect individual behaviors. Additionally, this study does not account for within-population heterogeneity, such as variations in demographics, socioeconomic factors, or regional policy enforcement, which may influence substance use patterns. Furthermore, confounding variables at the national level, including cultural attitudes toward substance use, healthcare access, and economic conditions, could impact both medical cannabis sales and consumption trends of other substances, potentially biasing the observed associations. While mixed effects models can account for random variation across units, they rely on the assumption that the random effects are uncorrelated with the regressors—a strong assumption that may not be plausible in our context, given the potential endogeneity between country-level characteristics and innovation outcomes. Instead, we opted for a FE specification, which allows us to control for unobserved time-invariant heterogeneity across countries that could confound the estimated relationships. To empirically justify this modeling choice, we performed a Hausman test which supported this choice.

### Summary and policy implications

This study demonstrates that legalization is associated in long-term growth in MC sales, serving as a proxy for innovation output and underscoring the critical role of regulatory frameworks in shaping the MC market. The findings indicate that MC demand is not only influenced by economic factors but also by social attitudes and interactions with other substance use behaviors. The observed substitution effect with tobacco consumption suggests that as MC becomes more accessible, a portion of consumers may reduce tobacco use. Conversely, the strong complementarity with cannabis consumption indicates that MC markets expand in response to broader societal acceptance and increased utilization of cannabis products. Furthermore, the event study results suggest that the effects of legalization are not transitory; rather, they drive sustained market expansion, with notable growth persisting in the long term.

The frequency and mode of cannabis consumption significantly influence substitution patterns and co-use with alcohol, tobacco, and other substances. Daily use of high-potency cannabis has been associated with a fivefold increase in the risk of psychosis, whereas less frequent use exhibits weaker associations (Hoch et al. 2024). Additionally, cannabis use disorder (CUD) affects 22% of those who use it, with prevalence rising to 33% among weekly or daily consumers (Hoch et al. 2024). The method of administration also plays a crucial role; oral cannabis consumption results in prolonged THC effects, while inhalation leads to shorter but immediate impairment (Hoch et al. 2024). Frequent users may develop tolerance, which in turn influences patterns of co-use (Hoch et al. 2024). Moreover, simultaneous use of cannabis and alcohol intensifies impairment, while the strong correlation between tobacco and cannabis use complicates risk assessments (Hoch et al. 2024). These findings highlight the importance of considering frequency and administration methods when evaluating cannabis-related health outcomes.

Given these insights, regulatory strategies should be tailored to the specific economic conditions and cultural perceptions of cannabis in each national context. Countries that have legalized MC should allocate resources to regulatory oversight and public health initiatives to mitigate unintended consequences, such as shifts in substance use behaviors. The evidence also suggests that a well-regulated MC market can generate sustained economic benefits, emphasizing the need for comprehensive legal frameworks that address licensing, production standards, and access pathways. Additionally, removing barriers to access and enhancing consumer education will support the development of a responsible and sustainable market. Future research should further explore macroeconomic conditions and behavioral dynamics that shape MC demand, enabling policymakers to refine strategies that balance economic growth with public health priorities. 

## Data Availability

I here with formally declare that the authors of this (“Manuscript”) have written the submitted dissertation independently. We did not use any outside support except for the quoted literature, databases and other sources mentioned in the paper. I clearly marked and separately listed all the literature and all of the other sources which I employed when producing this academic work, either literally or in content.
